# Organic zinc with moderate chelation strength enhances zinc absorption in the small intestine and expression of related transporters in the duodenum of broilers

**DOI:** 10.3389/fphys.2022.952941

**Published:** 2022-07-22

**Authors:** Yun Hu, Chuanlong Wang, Wei Wu, Yicheng Qu, Weiyun Zhang, Ding Li, Ling Zhu, Feiyu Gao, Bingxin Wu, Liyang Zhang, Xiaoyan Cui, Tingting Li, Yanqiang Geng, Xugang Luo

**Affiliations:** ^1^ Poultry Mineral Nutrition Laboratory, College of Animal Science and Technology, Yangzhou University, Yangzhou, China; ^2^ Institute of Animal Science, Chinese Academy of Agricultural Sciences, Beijing, China

**Keywords:** zinc absorption, the organic zinc with moderate chelation strength, zinc transporter, amino acid transporter, duodenum, broiler

## Abstract

Our previous study demonstrated that the absorption of zinc (Zn) from the organic Zn proteinate with moderate chelation strength was significantly higher than that of Zn from the inorganic Zn sulfate in the *in situ* ligated duodenal segment of broilers, but the underlying mechanisms are unknown. The present study aimed to determine the effect of organic Zn with moderate chelation strength and inorganic Zn on the Zn absorption in the small intestine and the expression of related transporters in the duodenum of broilers. The Zn-deficient broilers (13 days old) were fed with the Zn-unsupplemented basal diets (control) containing 25.72 and 25.64 mg Zn/kg by analysis or the basal diets supplemented with 60 mg Zn/kg as the Zn sulfate or the Zn proteinate with moderate chelation strength (Zn-Prot M) for 26 days. The results showed that the plasma Zn contents from the hepatic portal vein of broilers at 28 days and 39 days of age were increased (*p* < 0.05) by Zn addition and greater (*p* < 0.05) in the Zn-Prot M than in the Zn sulfate. On d 28, Zn addition upregulated (*p* < 0.05) mRNA expression of zinc transporter 1 (ZnT1), Zrt-irt-like protein 5 (ZIP5), y + L-type amino transporter 2 (y + LAT2) and b^0,+^-type amino acid transporter (rBAT), zinc transporter 4 (ZnT4) protein expression, and zinc transporter 9 (ZnT9) mRNA and protein expression in the duodenum. Moreover, ZnT9 mRNA expression, ZnT4, ZIP5, and rBAT protein expression, zinc transporter 7 (ZnT7), and y + LAT2 mRNA and protein expression in the duodenum of broilers on 28 days were higher (*p* < 0.05) in the Zn-Prot M than in the Zn sulfate. On d 39, supplemental Zn increased (*p* < 0.05) peptide-transporter 1 (PepT1) mRNA expression and y + LAT2 protein expression, while the mRNA expression of ZnT7 and Zrt-irt-like protein 3 (ZIP3) were higher (*p* < 0.05) for the Zn-Prot M than for the Zn sulfate in the duodenum. It was concluded that the Zn-Prot M enhanced the Zn absorption in the small intestine partially via upregulating the expression of ZnT4, ZnT7, ZnT9, ZIP3, ZIP5, y + LAT2, and rBAT in the duodenum of broilers.

## Introduction

Zinc (Zn) is an essential trace element that plays a critical role in the physiological and biochemical processes of humans and animals (Vallee and Falchuk, 1993; Romeo et al., 2014). The Zn deficiency could lead to decreased feed consumption, reduced growth, impaired skin-barrier function, diarrhea, and impaired immune (Muhamed and Vadstrup, 2014; Livingstone, 2015). Therefore, Zn is often added to broiler diets in the form of supplements to prevent Zn deficiencies or meet animal growth and production.

Traditionally, Zn is often added to the diets of broilers as the inorganic Zn sulfate. However, the absorption and utilization of Zn as the inorganic Zn sulfate in feedstuffs are very low in livestock and poultry. The unabsorbed Zn was discharged into the soil and river, which causes environmental pollution. Therefore, in recent years, organic Zn sources have been developed as alternatives to traditional inorganic Zn sources (Hill et al., 1987a; Hill et al., 1987b; Huang et al., 2009; Yu et al., 2017). Star et al. (2012) reported that the organic Zn amino acid complex was more available than Zn sulfate as reflected by the Zn contents in the tibia of broilers. De Grande et al. (2020) observed an increased apparent absorbability of Zn amino acid complex compared to Zn sulfate in young broilers. Studies from our laboratory have shown that the bioavailabilities of organic Zn sources for broilers are closely related to their chelation strengths [defined as the quotient of formation (**Q**_**f**_)] between Zn and their ligands (Huang et al., 2009; Yu et al., 2017). The absorption of Zn from organic Zn sources was significantly higher than that of Zn from the inorganic Zn source in the duodenum of broilers (Yu et al., 2017), and the organic Zn source with the moderate Q_f_ was more available than the organic Zn sources with weak or strong Q_f_ to the broilers (Huang et al., 2009). However, the underlying molecular mechanisms of the absorption of the organic Zn with moderate chelation strength in the duodenum of broilers remain unclear.

The mechanism of the inorganic Zn absorption has been well investigated in the mammalian intestine (Hennigar and McClung, 2018). The Zrt-irt-like proteins (ZIPs) and Zn transporters (ZnTs) are the two major families of Zn transport proteins involved in Zn transport in intestinal epithelial cells (Bin et al., 2018). There are 14 ZIP and 10 ZnT family members that have been identified as major contributors to the Zn transport in humans (Bin et al., 2018). The ZIPs increase the intracellular Zn concentrations by Zn import, and ZnTs reduce cytoplasmic Zn concentrations by Zn export (Bin et al., 2018). Some previous studies indicated that Zn transport proteins might also participate in the absorption of Zn as organic Zn sources in the small intestine (He et al., 2019; Diao et al., 2021). He et al. (2019) reported that organic Zn increased the mRNA expression of Zn transporter 1 (ZnT1) in the jejunum of broilers challenged with *Eimeria maxima* and *Clostridium perfringens*. Diao et al. (2021) reported that dietary supplementation with the organic Zn, particularly Zn lactate, significantly increased the mRNA expression of the jejunal Zn transporter 2 (ZnT2) in weaned piglets. In addition, the previous study has shown that the organic mineral complex could be absorbed in its intact form (Ashmead, 2001). Also, a previous study has revealed that the amino acid transporters might participate in the absorption of copper amino acid, complex in caco-2 cells (Gao et al., 2014). Several types of amino acid and peptide transporters, y^+^L-type amino transporter 1 (y^+^LAT1), L-type amino transporter 1 (LAT1), b^0,+^-type amino acid transporter (rBAT), B^0^-type amino acid transporter 1 (B^0^AT1) and peptide-transporter 1 (PepT1) were shown to participate in amino acid or peptide transport in the intestine (Dave et al., 2004; Fraga et al., 2005; Hu et al., 2008; Jando et al., 2017; Knöpfel et al., 2019; Rotoli et al., 2020). Our previous studies demonstrated that the Zn absorption in the *in situ* ligated duodenal segment of broilers was a saturable carrier-mediated process (Yu et al., 2008; Yu et al., 2017), while amino acid transporters LAT1 and B^0^AT1 might participate in the absorption of Fe as Fe amino acid chelates in the *in situ* ligated jejunum and ileum loops of broilers (Lu et al., 2018). However, no information is available regarding whether the aforementioned Zn, amino acid, and peptide transporters participate in the absorption of Zn as the organic Zn in the duodenum of broilers.

We hypothesized that the organic Zn source with moderate chelation strength would enhance the Zn absorption in the small intestine partially via up-regulating the expression of the aforementioned related transporters in the duodenum of broilers. Therefore, the objective of the present study was to determine the effect of supplemental organic Zn with moderate chelation strength and inorganic Zn on plasma Zn contents from the hepatic portal vein and gene expression of Zn, amino acid, and peptide transporters in the duodenum of broilers to test the aforementioned hypothesis.

## Materials and methods

### Experimental design and treatments

In the present study, a completely randomized design was adopted. A total of three dietary treatments were designed, including the Zn-unsupplemented corn-soybean meal basal diets (control, containing 25.72 and 25.64 mg Zn/kg by analysis) or the basal diets supplemented with 60 mg Zn/kg from the Zn sulfate (ZnSO_4_.7H_2_O) or the Zn proteinate with moderate chelation strength (Zn-Prot M, 17.09% Zn and Q_f_ = 51.6 by analysis), respectively. The dietary added Zn level was based on the Zn requirement (a total dietary Zn level of about 85 mg/kg) of broilers determined in our previous study (Huang et al., 2007b).

### Animals and diets

A total of 300 1-d-old Arbor Acres commercial male broilers (Jinghai Broiler Livestock Industry Group, Haimen, China) were fed a Zn-deficient starch-casein-based semi-purified diet (containing 17.69 mg Zn/kg of diet by analysis on an as-fed basis, Table 1) from d 1 to 13 to deplete the body Zn stores. At 13 days of age, after an overnight fast, a total of 240 broilers were weighed, selected, and randomly allotted to one of three treatments with eight replicate cages of 8 birds per cage. The broilers were maintained on a 24-h constant light schedule and handled in accordance with the Arbor Acres Broiler Management Guide (Aviagen, 2009), and allowed *ad libitum* access to diets and tap water containing no detectable Zn. Mortality was recorded daily, and body weight and feed intake per cage were measured on d 28 and 39 to calculate the average daily feed intake (ADFI), average daily gain (ADG), feed conversion rate (FCR, feed/gain), and mortality during d 14 to 28 and 29 to 39.

**TABLE 1:** Composition and nutrient levels of the basal diets for 1- to 39-day-old broilers (as-fed basis).

	D 1–21		D 22–39
Item	Semi-purified diet (d 1–13)	Corn–soybean meal diet (d 14–21)	Corn–soybean meal diet
Ingredients (%)			
Corn		53.82	59.16
Soybean meal		37.42	32.31
Corn starch	65.70		
Casein	25.75		
Cellulose	3.40		
Soybean oil		4.70	5.00
DL-Met	0.20	0.30	0.20
CaHPO_4_⋅2H_2_O^a^	1.60	1.87	1.65
CaCO_3_ ^a^	1.60	1.14	1.05
NaCl^a^	0.30	0.30	0.30
Micronutrients^b^	1.45 (containing macrominerals)	0.25	0.18
Corn starch + Zn^c^		0.20	0.15
Total	100.00	100.00	100.00
Nutrient levels			
ME, Kcal/kg	3140	3021	3096
CP^d^, %	22.72	21.74	19.61
Lys, %	1.80	1.12	1.00
Met, %	0.86	0.59	0.48
Met + cys, %	0.96	0.90	0.76
Ca^d^, %	0.99	1.02	0.89
Nonphytate P, %	0.48	0.45	0.40
Zn^d^, mg/kg	17.69	25.72	25.64

a Reagent grade.

b Provided per kilogram of diet: for d 1–13—vitamin A (all-trans retinol acetate), 12000 IU; vitamin D_3_ (cholecalciferol), 4500 IU; vitamin E (all-rac-α-tocopherol acetate), 33 IU; vitamin K_3_, 3 mg; vitamin B_1_, 3 mg; vitamin B_2_, 9.6 mg; vitamin B_6_, 4.5 mg; vitamin B_12_, 0.03 mg; pantothenic acid calcium, 15 mg; niacin, 54 mg; folic acid, 1.5 mg; biotin, 0.15 mg; choline, 700 mg; K (KCl), 3000 mg; Mg (MgSO_4_.7H_2_O), 600 mg; Cu (CuSO_4_⋅5H_2_O), 8 mg; Mn (MnSO_4_⋅H_2_O), 110 mg; Fe (FeSO_4_⋅7H_2_O), 40 mg; I (Ca(IO_3_)_2_·H_2_O), 0.35 mg; Se(Na_2_SeO_3_), 0.15 mg; for d 14–21—vitamin A (all-trans retinol acetate), 12000 IU; vitamin D3 (cholecalciferol), 4500 IU; vitamin E (all-rac-α-tocopherol acetate), 33 IU; vitamin K_3_, 3 mg; vitamin B_1,_ 3 mg; vitamin B_2_, 9.6 mg; vitamin B_6_, 4.5 mg; vitamin B_12_, 0.03 mg; pantothenic acid calcium, 15 mg; niacin, 54 mg; folic acid, 1.5 mg; biotin, 0.15 mg; choline, 700 mg; Cu (CuSO_4_⋅5H_2_O), 8 mg; Mn (MnSO_4_⋅H_2_O), 110 mg; Fe (FeSO_4_⋅7H_2_O), 40 mg; I (Ca(IO_3_)_2_·H_2_O), 0.35 mg; Se (Na_2_SeO_3_), 0.15 mg; for d 22–39—vitamin A (all-trans retinol acetate), 8000 IU; vitamin D_3_ (cholecalciferol), 3000 IU; vitamin E (all-rac-α-tocopherol acetate), 22 IU; vitamin K_3_, 2 mg; vitamin B_1_, 2 mg; vitamin B_2_, 6.4 mg; vitamin B_6_, 3 mg; vitamin B_12_, 0.02 mg; pantothenic acid calcium, 10 mg; niacin, 36 mg; folic acid, 1 mg; biotin, 0.1 mg; choline, 500 mg; Cu (CuSO_4_⋅5H_2_O), 8 mg; Mn (MnSO_4_⋅H_2_O), 80 mg; Fe (FeSO_4_⋅7H_2_O), 30 mg; I (Ca(IO_3_)_2_·H_2_O), 0.35 mg; Se(Na_2_SeO_3_), 0.15 mg.

c Zn supplements were added in place of equivalent weights of cornstarch.

d Determined values.

The starch–casein diet and corn–soybean meal basal diets were formulated to meet or exceed the nutrient requirements for broilers (NRC, 1994) except for Zn (Table 1). A single batch of basal diet was mixed and then divided into three aliquots, and the Zn sources were added to the basal diet according to the experimental treatments. Zinc sulfate (ZnSO_4_.7H_2_O) was reagent grade (Sinopharm Chemical Reagent Co. LTD., Shanghai, China) and contained 22.49% Zn by analysis (purity >99.5%). Zinc proteinate (feed grade) was provided by a commercial company (Alltech Inc., Nicholasville, KY, United States) and contained 17.09% Zn by analysis. The Q_f_ value of the Zn proteinate was analyzed to be 51.6, which is categorized as a moderate chelation strength according to the classification of Holwerda et al. (1995). The Zn proteinate contained AA (% of product) with lysine (1.33), methionine (0.15), threonine (0.93), aspartic acid (2.57), serine (1.29), glutamic acid (3.70), glycine (0.98), alanine (1.03), cysteine (0.89), valine (0.87), isoleucine (0.76), leucine (1.57), Tyrosine (0.78), phenylalanine (1.60), histidine (0.64), arginine (1.75), and proline (1.06) on a basis of analysis. Variable small amounts of l-lysine monohydrochloride or dl-methionine were added to the respective experimental diets according to the amounts of lysine and methionine from the supplemental organic Zn proteinate so as to balance lysine and methionine in each experimental diet. The analyzed Zn concentrations in diets are listed in Table 2.

**TABLE 2:** Analyzed Zn concentrations in diets of broilers from 14 to 39 days of age^a^.

Zn source	Added Zn, mg/kg	Analyzed Zn contents (d 14–21), mg/kg	Analyzed Zn contents (d 22–39), mg/kg
Control	0	25.72	25.64
Zn sulfate	60	84.91	85.27
Zn-Prot M^2^	60	86.93	84.29

a Values of analyzed Zn contents are based on triplicate determinations.

b Zn-Prot M = Zn proteinate with moderate chelation strength (Q_f_ = 51.6).

### Sample collections and preparations

The feed ingredients and diet samples from all the treatments were collected and analyzed for CP, Ca, and Zn before the initiation of the experiment to confirm CP, Ca, and Zn contents in diets. The Zn sulfate and the tap water were sampled for Zn analysis. The Zn proteinate was sampled for analyses of Zn, AA, and Q_f_.

At both 28 and 39 days of age, 24 broilers (3 chickens from each replicate cage) from each treatment were selected based on the average body weight of the cage, and anesthetized by intravenous injections of propofol (20 mg/kg body weight) via a wing vein. The blood sample was collected from the hepatic portal vein, and plasma was separated and stored at −20°C for analysis of Zn content. After blood collection, the birds were killed by cervical dislocation, the duodenum was separated and rinsed with ice-cold saline solution, and the intestinal mucosa was scraped with an ice-cold microscope slide, immediately frozen in liquid nitrogen, and then stored at −80°C for analyses of mRNA and protein expression. All samples from 3 birds in each replicate cage were pooled into one sample in equal ratios before analyses.

### Measurements of Zn, Ca, CP concentrations and Q_f_ value, and AA contents of the Zn proteinate

The concentrations of Zn in Zn sources, water, diets, and plasma, and Ca in feed ingredients or diets were determined by the 5110 ICP-OES (Agilent Technologies Australia (M) Pty Ltd., Australia) after wet digestions with HNO_3_ and HCIO_4_ as described previously (Huang et al., 2009). Validation of the Zn and Ca analyses were conducted using yellow soybean powder (GBW 10013 (GSB-4), National Research Center of Standard Materials, Beijing, China) and pork liver powder (GBW 10051 (GSB-29), National Research Center of Standard Materials, Beijing, China) as standard references. The CP concentrations in the feed ingredients and diet samples were determined using the Association of Official Analytical Chemists (1990) methods. The Q_f_ value of the Zn proteinate was determined by the 844 Professional VA (Metrohm Herisau, Switzerland) using a polarography method as described before (Huang et al., 2009; Luo et al., 2021). Amino acid contents in the Zn proteinate were analyzed using the L-8900 Amino Acid Analyzer (Hitachi High-Technologies Corporation, Tokyo, Japan).

### Quantitative real-time PCR

Total RNA was isolated from 50 mg of duodenal mucosa sample using 1 ml of TRIzol reagent (Life Technologies) and then treated with RNase-free DNase and reverse-transcribed to cDNA using a reverse transcription kit (Vazyme Biotech Co., Ltd.). Two microliters of diluted cDNA (1:20, vol/vol) were used for real-time PCR which was performed with applied biosystems quantstudio 3D digital PCR System (Applied Biosystems). All primers were listed in Table 3. Relative mRNA expression was normalized using *β-actin* and *GAPDH* as internal reference genes. Data were calculated using the 2^−ΔΔCt^ method (Livak and Schmittgen, 2001).

**TABLE 3:** Primer sequences for real-time PCR amplification.

Gene	GenBank ID	Primer sequence	Product length (bp)
*ZnT1*	XM_421021.5	F: 5′-AGA​GCC​TGG​GTT​TGG​ATT​CG-3′	229
		R: 5′-AGC​CCA​TGC​ATG​AAC​ACT​GA-3′	
*ZnT4*	XM_423325	F: 5′-GCC​ATC​TTG​ACG​GAC​GTA​GT-3′	190
		R: 5′-CAA​GGT​ACA​CCA​GGA​CAC​CC-3′	
*ZnT5*	NM_001031419.2	F: 5′-ATG​GAG​GAA​AAG​TAC​AGC​AGC​C-3′	118
		R: 5′-TCA​GAA​ACT​TGG​CGA​AGC​AC-3′	
*ZnT7*	NM_001008788.1	F: 5′-TGC​TGC​CCC​TCT​CCA​TTA​AG-3′	114
		R: 5′-AGA​GGT​TGC​GGG​ATG​TCT​TG-3′	
*ZnT9*	XM_015285471.1	F: 5′-ACA​TGT​TTT​TCC​GTG​CAG​CC-3′	265
		R: 5′-CGG​AAC​ACA​ACC​TTT​ACC​AGC-3′	
*ZnT10*	XM_015283897.1	F: 5′-GAG​CTG​TAG​AGA​TGG​GTC​GC-3′	184
		R: 5′-ACA​CCG​AGC​AAA​CCG​ATG​AT-3′	
*ZIP3*	XM_015299966.2	F: 5′-CAT​ACA​TCC​AGG​AGG​CAG​AGG-3′	246
		R: 5′-CCT​GGA​TGA​TCT​TGA​CGG​GG-3′	
*ZIP5*	XM_025145573.1	F: 5′-CCA​AGA​TGA​AAC​GCA​CGC​AA-3′	284
		R: 5′-TAA​GCT​GCG​ACC​AAG​TCC​TG-3′	
*B* ^*0*^ *AT1*	XM_419056.6	F: 5′-CTT​GGG​TGA​GGT​AGG​TGG​GA -3′	167
		R: 5′-GAT​GCG​GGT​GCT​CTC​ATG​TA-3′	
*LAT1*	NM_001030579.2	F: 5′-GGA​AAG​GCC​CAT​CAA​GGT​GA-3′	248
		R: 5′-ACG​ATT​CTT​GGG​GAA​CCA​C-3′	
*y + LAT2*	XM_001231336.4	F: 5′-ATC​TTG​CGC​CTG​AGG​GAA​GG-3′	296
		R: 5′-CGG​CTC​TGA​ACT​CCA​ATC​TGT-3′	
*rBAT*	XM_004935370.3	F: 5′-CCT​AGG​AGG​AGA​GGC​ACG​AA-3′	223
		R: 5′-TCC​TGC​ATA​GGG​CTG​CAA​TG-3′	
*EAAT3*	XM_424930.6	F: 5′-CGT​CCA​GGC​CTG​TTT​TCA​AC-3′	171
		R: 5′-TCG​GAA​TAC​ATG​CCC​ACG​AT-3′	
*PepT1*	NM_204365.1	F: 5′-AAA​ACA​GGT​TTC​GGC​ATC​GC-3′	167
		R: 5′-CTG​CTG​GTC​AAA​AAG​TGC​CC-3′	
*β-actin*	NM_205518.1	F: 5′-CGG​TAC​CAA​TTA​CTG​GTG​TTA​GAT​G-3′	163
		R: 5′-GCC​TTC​ATT​CAC​ATC​TAT​CAC​TGG-3′	
*GAPDH*	NM_204305.1	F: 5′-CTT​TGG​CAT​TGT​GGA​GGG​TC-3′	128
		R: 5′-ACG​CTG​GGA​TGA​TGT​TCT​GG-3′	

*ZnT1*, zinc transporter 1; *ZnT4*, zinc transporter 4; *ZnT5*, zinc transporter 5; *ZnT7*, zinc transporter 7; *ZnT9*, zinc transporter 9; *ZIP3*, Zrt-irt-like protein 3; *ZIP5*, Zrt-irt-like protein 5; *B* ^*0*^ *AT1*, B-0-system neutral amino acid co-transporter; *LAT1*, L-type amino acid transporter 1; *y + LAT2*, y + L-type amino acid transporter 2; *rBAT*, b0,+-type amino acid transporter; *EAAT3*, excitatory amino acid transporter 3; *GAPDH*, glyceraldehyde-3-phosphate dehydrogenase; F, forward; R, reverse.

### Total protein extraction and Western blotting

Total protein was extracted from 50 mg of duodenal mucosa sample as previously described (Bai et al., 2021). The protein concentration was determined according to the manufacturer’s instructions of the Pierce BCA Protein Assay kit (Rockford, IL). Forty or 60 μg of protein were used for electrophoresis on a 7.5 or 10% SDS-PAGE gel. Western blotting analyses for rabbit anti-human ZnT1 (ab214356, Abcam, diluted 1:1,000), rabbit anti-mouse ZnT4 (BA3484, Boster, diluted 1:1,000), rabbit anti-human ZnT5 (25604-1-AP, Proteintech, diluted 1:1,000), rabbit anti-human ZnT7 (13966-1-AP, Proteintech, diluted 1:1,000), rabbit anti-human ZnT9 (BS62531, Bioworld, diluted 1:1,000), rabbit anti-human ZnT10 (ab229954, Abcam, diluted 1:1,000), rabbit anti-human ZIP3 (ab254868, Abcam, diluted 1:500), rabbit anti-human ZIP5 (ab105194, Abcam, diluted 1:1,000), rabbit anti-human B^0^AT1 (ab180516, Abcam, diluted 1:1,000), rabbit anti-human LAT1 (DF8065, Affinity Biosciences, diluted 1:500), rabbit anti-human y + LAT2 (13823-1-AP, Proteintech, diluted 1:1,000), rabbit anti-human rBAT (DF7379, Affinity Biosciences, diluted 1:1,000) and rabbit anti-human PepT1 (A03672-1, Boster, diluted 1:500) were carried out according to the recommended protocols provided by the manufacturers. We used the goat anti-rabbit secondary antibody (ab7090, Abcam, diluted 1:10,000) in the case of ZnT1, ZnT4, ZnT5, ZnT7, ZnT9, ZnT10, ZIP3, ZIP5, B^0^AT1, LAT1, y + LAT2, rBAT, and PepT1. All primary antibodies used in the present study were human/mouse-derived antibodies purchased from commercial companies. Images were recorded with a chemiluminescence image scanner (Tanon, Shanghai, China) and the band density was analyzed with Tanon Gis 1D software (Tanon, Shanghai, China). Data were presented as the ratio of ZnT1, ZnT4, ZnT5, ZnT7, ZnT9, ZnT10, ZIP3, ZIP5, B^0^AT1, LAT1, y + LAT2, rBAT, or PepT1 protein band intensity to β-tubulin protein band intensity. The β-tubulin protein was used to normalize the expression levels of target proteins.

### Statistical analyses

All data were analyzed by one-way ANOVA using the general linear model procedures of SAS 9.4 (SAS Institute Inc., Cary, NC). Differences among means were tested by the LSD method. Percentage data for the mortality of birds were transformed to arcsine for analysis. The replicate cage was the experimental unit, and the statistical significance was set at *p <* 0.05.

## Results

### Growth performance and mortality

The Zn source did not affect (*p* > 0.14) the ADG, ADFI, FCR, and mortality of broilers during d 14 to 28 and d 29 to 39 of age (Table 4).

**TABLE 4:** Effect of dietary Zn source on growth performance and mortality of broilers^a^.

Zn source	D 14–28				D 29–39			
	ADG (g/d)	ADFI (g/d)	FCR (g/g)	Mortality^b^	ADG (g/d)	ADFI (g/d)	FCR (g/g)	Mortality^b^
Control	36.1	51.0	1.42	0.00	73.6	113	1.54	1.56
Zn sulfate	37.4	52.1	1.39	0.00	72.3	114	1.58	0.00
Zn-Prot M	37.8	52.7	1.42	1.56	75.6	118	1.57	0.00
SEM	0.4	0.4	0.01	0.52	0.7	1	0.01	0.52
*p*-value	0.148	0.267	0.350	0.385	0.160	0.197	0.558	0.385

ADG, average daily gain; ADFI, average daily feed intake; FCR, feed conversion rate (feed/gain); Zn-Prot M, Zn proteinate with moderate chelation strength (Q_f_ = 51.6).

a Values are the means of 7/8 replicate cages of four to eight birds per replicate cage (n = 7/8).

b Percentage data for the mortality of birds were transformed to arcsine for analysis.

### Zn content in plasma from the hepatic portal vein

The Zn contents in plasma from the hepatic portal vein of broilers at 28 d and 39 days of age were affected (*p* < 0.0001) by the Zn source (Table 5). Compared with the control, the addition of Zn increased (*p* < 0.05) Zn contents in the plasma from the hepatic portal vein of broilers at 28 d and 39 days of age. Furthermore, Zn contents in plasma from the hepatic portal vein of broilers at 28 d and 39 days of age were higher (*p* < 0.05) for the Zn-Prot M than for the Zn sulfate.

**TABLE 5:** Effect of dietary Zn source on Zn content in plasma from the hepatic portal vein of 28- and 39-day-old chickens^a^.

Zn source	Zn contents in plasma, ug/mL	
	D 28	D 39
Control	1.22^c^	1.21^c^
Zn sulfate	1.65^b^	1.72^b^
Zn-Prot M	1.89^a^	1.97^a^
SEM	0.08	0.08
*p*-value	<0.0001	<0.0001

Zn-Prot M, Zn proteinate with moderate chelation strength (Q_f_ = 51.6).

^a,b,c^Means with different superscripts within the same column differ significantly (*p* < 0.05).

a Values are the means of 7/8 replicate cages of 3 birds per replicate cage (*n* = 7/8).

### mRNA expression levels of Zn, amino acid, and peptide transporters

**FIGURE 1:**
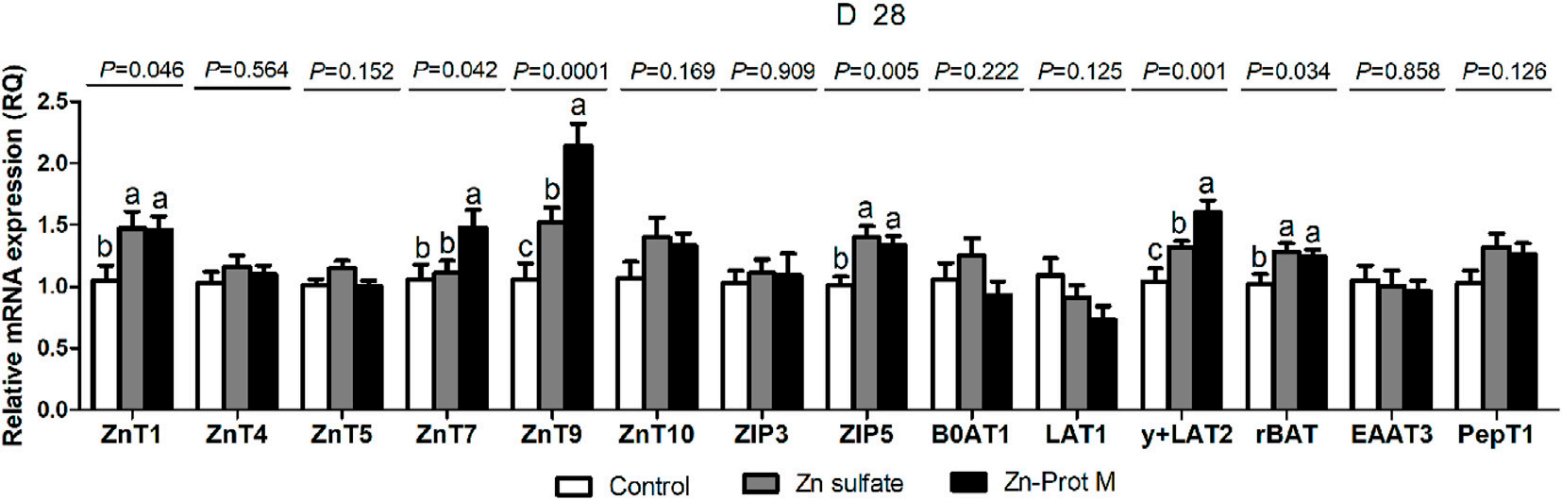
Effect of dietary Zn source on the mRNA expression levels of Zn, amino acid, and small peptide transporters in the duodenum of broilers at 28 days of age. Values are means ± SE, (*n* = 7/8). Lacking the same letters (a, b, and c) means significant differences, *p* < 0.05. ZnT1, zinc transporter 1; ZnT4, zinc transporter 4; ZnT5, zinc transporter 5; ZnT7, zinc transporter 7; ZnT9, zinc transporter 9; ZIP3, Zrt-irt-like protein 3; ZIP5, Zrt-irt-like protein 5; B0AT1, B-0-system neutral amino acid co-transporter; LAT1, L-type amino acid transporter 1; y + LAT2, y + L-type amino acid transporter 2; rBAT, b0,+-type amino acid transporter; EAAT3, excitatory amino acid transporter 3; PepT1, peptide-transporter 1; Zn-Prot M, Zn proteinate with moderate chelation strength (Q_f_ = 51.6). The mRNA expression levels were calculated as the relative quantities (RQs) of the target gene mRNA to the geometric mean of *β-actin* and *GAPDH* mRNA using the 2^−ΔΔCT^ method.

The Zn source did not affect (*p* > 0.12) the mRNA expression levels of ZnT4, ZnT5, ZnT10, ZIP3, B^0^AT1, LAT1, EAAT3, and PepT1 but influenced (*p* < 0.05) the mRNA expression levels of ZnT1, ZnT7, ZnT9, ZIP5, y + LAT2, and rBAT in the duodenum of broilers at 28 days of age (Figure 1). Two Zn sources up-regulated (*p* < 0.05) the mRNA expression levels of ZnT1, ZnT9, ZIP5, y + LAT2, and rBAT in the duodenum of broilers at 28 days of age compared with the control. Compared with the Zn sulfate, dietary supplementation with Zn-Prot M increased (*p* < 0.05) the ZnT7, ZnT9, and y + LAT2 mRNA expression levels in the duodenum of broilers at 28 days of age.

**FIGURE 2:**
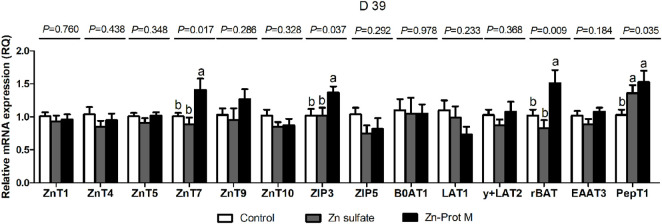
Effect of dietary Zn source on the mRNA expression levels of Zn, amino acid, and small peptide transporters in the duodenum of broilers at 39 days of age. Values are means ± SE, (n = 7/8). Lacking the same letters (a and b) means significant differences, *p* < 0.05. ZnT1, zinc transporter 1; ZnT4, zinc transporter 4; ZnT5, zinc transporter 5; ZnT7, zinc transporter 7; ZnT9, zinc transporter 9; ZIP3, Zrt-irt-like protein 3; ZIP5, Zrt-irt-like protein 5; B0AT1, B-0-system neutral amino acid co-transporter; LAT1, L-type amino acid transporter 1; y + LAT2, y + L-type amino acid transporter 2; rBAT, b0,+-type amino acid transporter; EAAT3, excitatory amino acid transporter 3; PepT1, peptide-transporter 1; Zn-Prot M, Zn proteinate with moderate chelation strength (Q_f_ = 51.6). The mRNA expression levels were calculated as the relative quantities (RQs) of the target gene mRNA to the geometric mean of *β-actin* and *GAPDH* mRNA using the 2^−ΔΔCT^ method.

The Zn source had no effect (*p* > 0.18) on the mRNA expression levels of ZnT1, ZnT4, ZnT5, ZnT9, ZnT10, ZIP5, B^0^AT1, LAT1, y + LAT2, and EAAT3 in the duodenum of broilers at 39 days of age (Figure 2). The mRNA expression levels of ZnT7, ZIP3, rBAT, and PepT1 in the duodenum of broilers at 39 days of age were affected (*p* < 0.05) by the Zn source. Compared with the control, two Zn sources increased (*p* < 0.05) the mRNA expression levels of PepT1 in the duodenum of broilers at 39 days of age with no difference (*p* > 0.43) between the two Zn sources. Moreover, the mRNA expression levels of ZnT7, ZIP3, and rBAT in the duodenum of broilers on d 39 were higher (*p* < 0.05) for the Zn-Prot M than for the control and Zn sulfate with no difference (*p* > 21) between the control and Zn sulfate.

### Protein expression levels of Zn, amino acid, and peptide transporters

**FIGURE 3:**
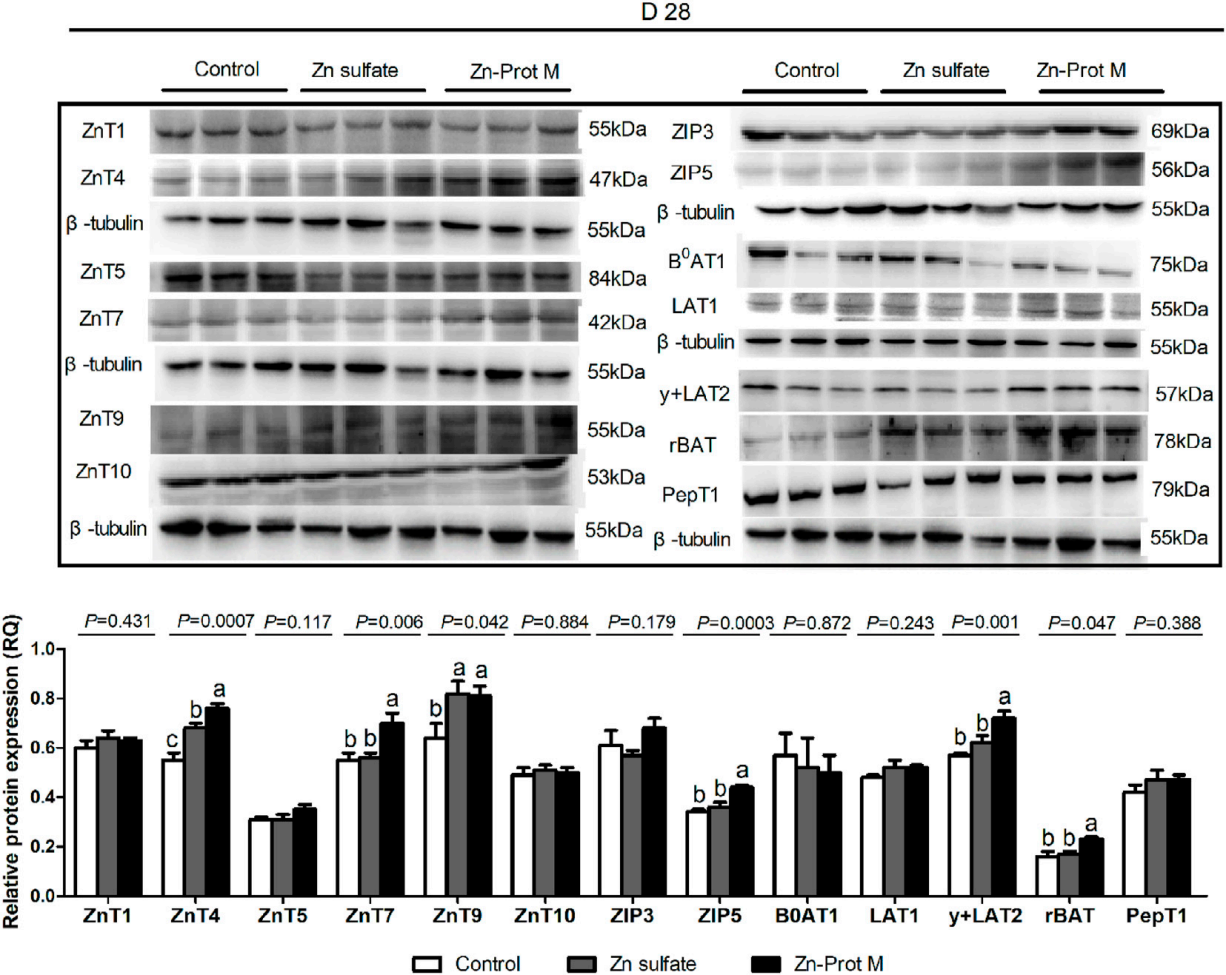
Effect of dietary Zn source on the protein expression levels of Zn, amino acid, and small peptide transporters in the duodenum of broilers at 28 days of age. Values are means ± SE, (n = 7/8). Lacking the same letters (a, b, and c) means significant differences, *p* < 0.05. ZnT1, zinc transporter 1; ZnT4, zinc transporter 4; ZnT5, zinc transporter 5; ZnT7, zinc transporter 7; ZnT9, zinc transporter 9; ZIP3, Zrt-irt-like protein 3; ZIP5, Zrt-irt-like protein 5; B0AT1, B-0-system neutral amino acid co-transporter; LAT1, L-type amino acid transporter 1; y + LAT2, y + L-type amino acid transporter 2; rBAT, b0,+-type amino acid transporter; PepT1, peptide-transporter 1; Zn-Prot M, Zn proteinate with moderate chelation strength (Q_f_ = 51.6). The protein expression levels were calculated as the relative quantities (RQs) of the target gene protein to the β-tubulin protein.

The Zn source did not affect (*p* > 0.11) protein expression levels of both ZnT1, ZnT5, ZnT10, ZIP3, B^0^AT1, LAT1, and PepT1, but influenced (*p* < 0.05) the protein expression levels of ZnT4, ZnT7, ZnT9, ZIP5, y + LAT2, and rBAT in the duodenum of broilers at 28 days of age (Figure 3). Compared with the control, two Zn sources increased (*p* < 0.05) the protein expression levels of ZnT4 and ZnT9 in the duodenum of broilers for 28 days. Compared with the Zn sulfate, the Zn-Prot M increased (*p* < 0.05) the ZnT4 protein expression, but there was no difference (*p* > 0.80) in the ZnT9 protein expression between the two Zn sources in the duodenum of broilers on 28 days. Furthermore, the protein expression levels of ZnT7, ZIP5, y + LAT2, and rBAT in the duodenum of broilers on d 28 were higher (*p* < 0.05) for the Zn-Prot M than for the control and Zn sulfate with no difference (*p* > 0.08) between the control and Zn sulfate.

**FIGURE 4:**
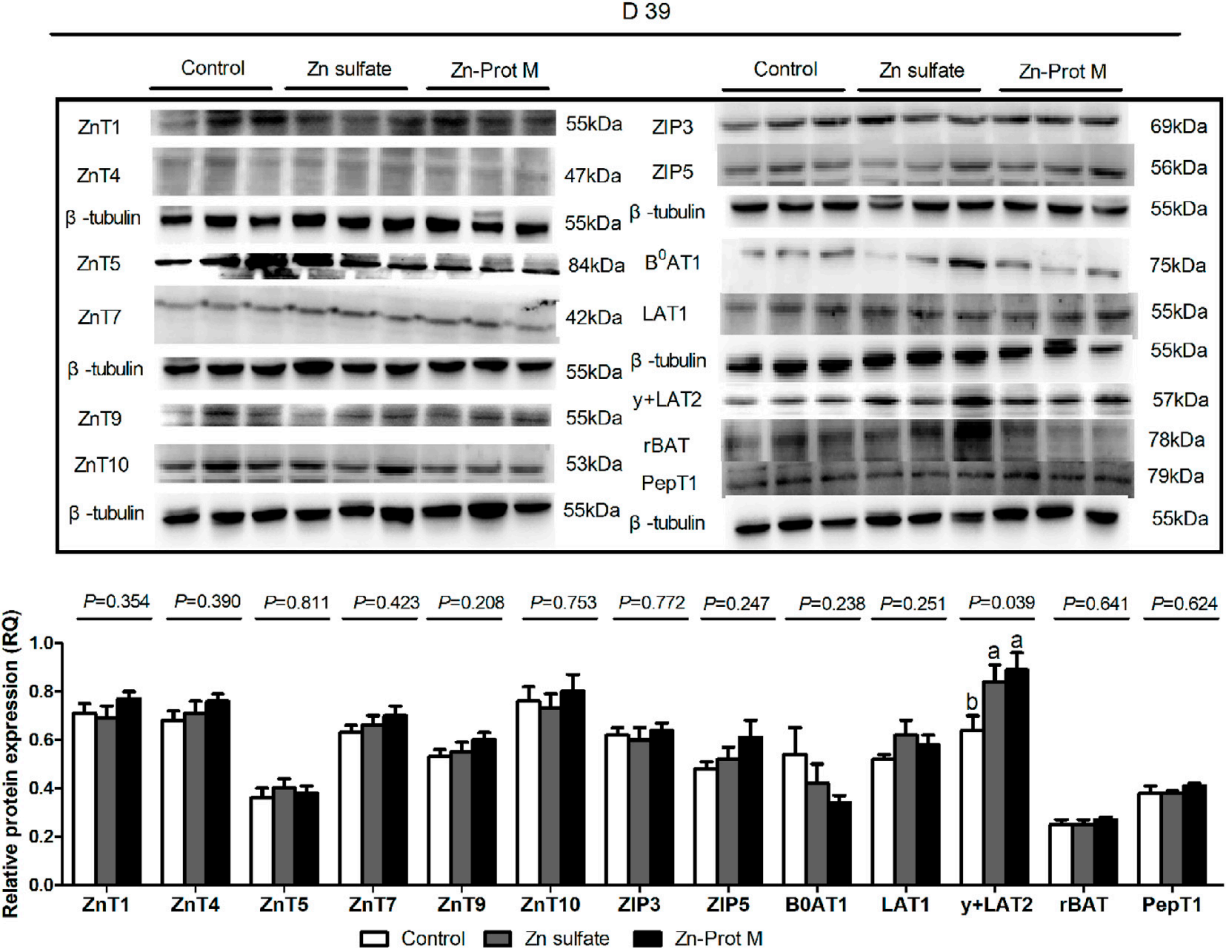
Effect of dietary Zn source on the protein expression levels of Zn, amino acid, and small peptide transporters in the duodenum of broilers at 39 days of age. Values are means ± SE, (n = 7/8). Lacking the same letters (a and b) means significant differences, *p* < 0.05. ZnT1, zinc transporter 1; ZnT4, zinc transporter 4; ZnT5, zinc transporter 5; ZnT7, zinc transporter 7; ZnT9, zinc transporter 9; ZIP3, Zrt-irt-like protein 3; ZIP5, Zrt-irt-like protein 5; B0AT1, B-0-system neutral amino acid co-transporter; LAT1, L-type amino acid transporter 1; y + LAT2, y + L-type amino acid transporter 2; rBAT, b0,+-type amino acid transporter; PepT1, peptide-transporter 1; Zn-Prot M, Zn proteinate with moderate chelation strength (Qf = 51.6). The protein expression levels were calculated as the relative quantities (RQs) of the target gene protein to the β-tubulin protein.

The Zn source had no effect (*p* > 0.20) on the protein expression levels of ZnT1, ZnT4, ZnT5, ZnT7, ZnT9, ZnT10, ZIP3, ZIP5, B^0^AT1, LAT1, rBAT, and PepT1 in the duodenum of broilers at 39 days of age (Figure 4). The protein expression of y + LAT2 in the duodenum of broilers for 39 days was affected (*p* < 0.05)the by Zn source. Compared with the control, two Zn sources up-regulated (*p* < 0.05) the y + LAT2 protein expression in the duodenum of broilers on d 39. However, no difference (*p* > 0.05) was observed for the y + LAT2 protein expression between the Zn-Prot M and Zn sulfate.

## Discussion

The results from the present study have demonstrated that the Zn-Prot M significantly and consistently increased both Zn contents in plasma from the hepatic portal vein of broilers and mRNA and/or protein expression of the ZnT4, ZnT7, ZnT9, ZIP3, ZIP5, y + LAT2, and rBAT in the duodenum of broilers, suggesting that the Zn-Prot M could enhance the Zn absorption in the small intestine partially via up-regulating the expression of the aforementioned transporters in the duodenum of broilers, which has supported our hypothesis. The aforementioned findings have been not reported before and provided scientific bases for developing and applying the more absorbable new organic Zn with moderate chelation strength in the broiler production and also a new insight into developing strategies to further improve the Zn absorption in the small intestine of broilers in the future.

Dietary Zn is taken up from the lumen into intestinal-mucosal cells and subsequently transported across the basolateral membrane to the portal blood of the liver (Smith et al., 1979). Thus, Zn content in plasma of the hepatic portal vein was a sensitive index in reflecting differences in Zn absorption from different Zn sources. In the present study, the birds were depleted of body Zn stores from d 1 to 13 to increase their sensitivity to Zn addition. A similar approach was used in our previous study on the Zn absorption in broilers (Yu et al., 2017). In the present study, we found that the Zn contents in plasma from the hepatic portal vein of broilers at 28 and 39 days of age were significantly higher for the Zn-Prot M than for the Zn sulfate. Our results have confirmed the previous observation that Zn content in the plasma of the hepatic portal vein of broilers was higher for the organic Zn with moderate chelation strength than for the Zn sulfate (Yu et al., 2017). Our findings along with those of others have clearly indicated that the organic Zn with moderate chelation strength had a higher Zn absorption in the small intestine of broilers.

The mechanisms of the absorption of the inorganic Zn in the small intestine have been well investigated. The Zn uptake in the intestine exhibits both a nonsaturable diffusive process and a saturable carrier-mediated process (Steel and Cousins, 1985; Tacnet et al., 1990). Our previous studies have demonstrated that the Zn absorption in the duodenum of broilers was via a saturable carrier-mediated pathway (Yu et al., 2008; Yu et al., 2017). In mammals, the ZIPs and ZnTs are responsible for Zn transport across membranes (Wang and Zhou, 2010). The ZnT transporters mediate the Zn efflux from the cytosol or sequestration into organelles/vesicles, whereas those of the ZIP proteins move Zn from extracellular or organellar/vesicular into the cytoplasm (Kambe et al., 2004). The ZIP family has 14 members (ZIP1 to ZIP14) and the ZnT family contains 10 members (ZnT1 to ZnT10). However, whether the above-mentioned Zn transporters participated in the absorption of Zn as organic Zn sources in the duodenum of broilers was unclear. It was reported that the organic Zn increased ZnT1 mRNA expression in the jejunum of broilers (He et al., 2019). However, in the present study, Zn supplementation, regardless of the Zn source, significantly increased ZnT1 mRNA expression in the duodenum of broilers at 28 days of age, but no difference was observed between the two Zn sources. The above disparities might be caused by different tissues, the dose of supplementation, or Zn sources used in these studies. In addition, in the present study, Zn addition significantly increased ZIP5 mRNA expression, ZnT4 protein expression, ZnT9 mRNA, and protein expression in the duodenum of broilers at 28 days of age. Moreover, ZnT4 and ZnT7 protein expression levels in the duodenum of broilers at 28 days of age were significantly higher for the organic Zn than for the inorganic Zn. The ZnT4 expression in the small intestine was restricted to the epithelium of the villus, and the expression of ZnT4 in enterocytes could promote Zn secretion into the bloodstream (Yu et al., 2007). The ZnT7 was predominantly located in the Golgi apparatus and involved in transporting the cytoplasmic Zn into the Golgi apparatus of the cell for Zn storage (Kirschke and Huang, 2003; Wang and Zhou, 2010). In a previous study, The Zn-deficient mice showed lower serum and tissue-associated Zn content and reduced body weight gain (Huang et al., 2007a). Thus, the authors concluded that ZnT7-mediated Zn incorporation into Golgi is essential for dietary Zn absorption. Therefore, increased ZnT4 and ZnT7 protein expression could enhance the absorption of Zn as the organic Zn.

A previous study indicated that the organic Zn (Zn histidine complex) could be absorbed via its intact form in the perfused rat intestine (Wapnir et al., 1983). In addition, Gao et al. (2014) reported that amino acids could promote copper absorption, and the enhanced transport of copper in amino acid complex appears to be mediated by amino acid transporters in the Caco-2 cell culture model. Furthermore, our previous study demonstrated that the amino acid transporters LAT1 and B^0^AT1 might be involved in the absorption of Fe as Fe amino acid chelates in the *in situ* ligated jejunum or ileum loops of broilers (Yu et al., 2008). Similar results have been observed in the present study that the protein expression levels of y + LAT2 and rBAT in the duodenum of broilers at 28 days of age were significantly higher for the Zn-Prot M than for the Zn sulfate, indicating that these amino acid transporters might be involved in the absorption of Zn from the organic Zn-Prot M.

However, in the present study, the post-transcriptional regulation of gene expression is complex, and the mismatches between the mRNA and protein expression of some of the above transporters were observed. For instance, the mRNA expression levels of the ZnT9 on d 28 and the ZnT7, ZIP3, and rBAT on d 39 in the duodenum were significantly enhanced by the addition of the organic Zn-Prot M compared to the inorganic Zn sulfate, while no significant changes were detected for their corresponding protein expression. The protein expression levels of ZnT4, ZIP5, and rBAT on d 28 in the duodenum were significantly up-regulated by the addition of the organic Zn-Prot M compared to the inorganic Zn sulfate, but no significant alterations were detected for their corresponding mRNA expression. The disparities in mRNA and protein expression of the above transporters might be due to the delay in protein synthesis, the proportion of translation, and the modification of protein after synthesis (Wang et al., 2011; Liu et al., 2016). It should be pointed out that the up-regulated expression of some Zn and amino acid transporters in the duodenum due to the addition of the organic Zn-Prot M could only partially explain the enhanced absorption of Zn as the organic Zn-Prot M in the small intestine of broilers. Therefore, further studies are necessary to address the gene expression of Zn, amino acid, and peptide transporters in the jejunum and ileum of broilers and their associations with the Zn absorption in the small intestine of broilers as affected by the above organic and inorganic Zn sources.

In conclusion, the organic Zn proteinate with moderate chelation strength enhanced the Zn absorption in the small intestine of broilers, and the ZnT4, ZnT7, ZnT9, ZIP3, ZIP5, y + LAT2, and rBAT might participate in the absorption of Zn as the Zn proteinate with moderate chelation strength in the duodenum of broilers.

## Data Availability

The original contributions presented in the study are included in the article/Supplementary Material; further inquiries can be directed to the corresponding author.
